# Supplementary material for: An International Inter-Consortium Validation of Knowledge-Based Plan Prediction Modeling for Whole Breast Radiotherapy Treatment
Source: Cancers (Basel). 2025 Nov 5;17(21):3576. doi: 10.3390/cancers17213576 (PMC12610825; doi:10.3390/cancers17213576)
Supplement: Supplementary file 1 [file cancers-17-03576-s001.zip › cancers-3869648-supplementary.pdf]

Supplementary Material S1 – Literature review of national or multi-institutional KB models experience

Recently, different national consortia have been organized, aiming to implement a national and shared KB model, built based on different institutes' single models. The literature for such national or multi-institutions experiences has increased in recent years.

One of the first experiences was in 2016, by Berry et al. [45] which evaluated the utility of knowledge-based planning (KBP) in identifying systematic variations in intensity modulated radiotherapy (IMRT) plans across multiple campuses of a single institution. A KBP model was developed from prior plans at the main campus (MC) and applied to patient plans at the main campus and four regional sites (RS) on 172 predicted DVHs of test patients. Results show that RS1 plans deviated the most from the model, while RS2 plans were closest to the model's expectations. RS3 plans exhibited variability but were comparable to MC plans. RS4 had insufficient data for significant analysis. Overall, KBP can highlight subtle differences in planning practices, aiding in improving consistency and quality of care across campuses.

In 2017, Schubert et al. [46] evaluated the effectiveness of a KB optimization process for volumetric modulated arc therapy in prostate cancer treatment across multiple institutions within the German RapidPlan Consortium (GRC). They built and trained an RP model using data from 43 patients, which was then shared among the GRC (six institutes plus an external site from a different country to increase the heterogeneity of the patient sampling). The model validation was performed comparing RP against reference plans optimized according to institutional procedures. A total of 60 patients from seven institutes were used. The multicentric validation showed that RP-based plans were generally consistent with manually optimized plans, with some modest improvements observed in certain dose regions. However, variations in performance were noted due to differences in contouring protocols and optimization emphasis. Overall, the study suggests that sharing models among clinical institutes in a cooperative framework is feasible for prostate cancer treatment optimization, although fine-tuning may be necessary depending on specific institutional protocols and priorities.

The Japanese group of Ueda et al. [47] evaluated a commercial knowledge-based planning system for prostate cancer treatment across five radiation therapy departments. Each institute provides an RP model and, to verify whether the models performed correctly, KBP was compared with manual optimization planning in two cases from one institute, considering the generated estimated doses based on geometric and dosimetric information. Results show no significant differences in organ at risk (OAR) and target volumes between manual and knowledge-based planning, but variations in dose distribution were observed between institutes. The study underscores the importance of verifying model performance across different institutes before sharing knowledge-based planning models.

In 2019, another Japanese group (Kamina et al. [48] investigated whether additional manual objectives are necessary for RapidPlan (RP) in head and neck cancer (HNC) treatment optimization across multiple institutes in Japan. They compared RP plans with and without manual objectives (M-RP plans) for oropharynx cancers, finding no major differences in planning target volume (PTV). However, M-RP plans achieved dose requirements for the brainstem and spinal cord more consistently, with upper objectives needed, while RP plans showed relatively higher achievement rates for parotid glands. The study concludes that M-RP plans do not need re-optimization, requiring only upper objectives for critical structures, while reducing parotid gland doses with auto-generated objectives.

In 2019, Kavabaugh et al. [49] assessed the feasibility of using a single-institution knowledge-based planning (KBP) model as a quality control tool for multi-institutional clinical trials, focusing on non-small cell lung cancer. They retrospectively evaluated its efficacy using plans from Radiation Therapy Oncology Group (RTOG) study 0617. Three other institutes provided 25 patient tests following RTOG 0617 guidelines. The KBP model significantly improved target coverage and organ at risk sparing compared to clinical trial data, identifying patients who would benefit from specific optimization objectives. Overall, KBP models can enhance plan quality and assist in making anatomy-based dosimetric trade-offs in clinical trials.

The same year, the Australian group (Panettieri et al. [27]) assessed the implementation and clinical impact of a collaborative automated planning model for prostate cancer treatment using Intensity-Modulated Radiation Therapy/Volumetric-Modulated Arc Therapy. The model, built with 110 clinically approved and treated prostate plans provided by different radiotherapy centers, aims to standardize treatment planning by minimizing variations due to contouring and dose protocols. To investigate its impact, a total of 126 patients, originally manually inverse planned (OP), were replanned using RP without additional planner manual intervention. Results show that the automated plans, compared to manually inverse planned ones, provided comparable or better outcomes for target and organ at risk metrics, with statistically significant reductions in low doses to the bladder and rectum. The study demonstrates the feasibility and potential benefits of using automated planning models across different institutions to improve treatment planning results and efficiency.

More recently, the MIKAPOCo Italian consortium, published (Tudda et al. [28]) a study that assessed inter-institute variability of knowledge-based (KB) models for right breast cancer patients undergoing whole breast irradiation. Ten institutions developed KB models using RapidPlan and tested them on 20 new patients, each exporting DVH predictions of heart, ipsilateral lung, contralateral lung, and contralateral breast. Inter-institute variability was quantified by the inter-institute of predicted DVHs' mean doses. Results show low variability in predicted doses for organs at risk, except for one institution. Models were generally transferrable across institutes, except for outliers. Findings support the potential for generating benchmarks for plan prediction incorporating inter-institute variability. Within the MIKAPOCo Italian consortium, a similar work performed by Tudda et al. has been performed in Castriconi et al. [50], considering the left breast cancer patient. Plan prediction performance was tested on 16 patients (2 patients per center) leading to a high transferability among models and low inter-institutional variability of 2% for plan prediction. PC1 analysis suggested no relevant difference among models, except for one center showing a systematic larger spring of the heart, concomitant to a worse PTV coverage, due to high priority in sparing the left anterior descending coronary artery.

Supplementary Material S2—Procedures for building KB models and estimates of goodness of regression (from Tudda et al. [7])

According to previously defined procedures and evidences [19,51], for every patient of each institute's data sets, a "virtual" plan geometry was set, using two "fake" arcs (60°–220° cw and ccw, collimator rotation of 10° and 350°, respectively) and associated with the tangential field clinical dose distribution. This procedure was found to better capture patient's geometry/anatomy characteristics, to improve prediction in the case of concave-shaped PTVs, and to be easily usable in the case of automatic plan optimization by KB templates, following the recently introduced ViTAT (virtual tangential field arc therapy) approach [10]. After completing this procedure, plans were used to train the models. During the training phase, the system correlates PC between 'real' DVH (coming from the tangential field dose map) and the 'fake' GEDVH based on arcs geometry. However, as the same fake arc geometry for configuration is used for the DVH prediction in a new patient, the relationship between geometric and dosimetric features has been maintained.
All available OARs were considered; consequently, prediction models for the ipsilateral lung were generated for all centers while models for the contralateral lung, the left breast, and the heart were generated only for those centers whose contouring included them. After the first extraction/training, Rapid Plan tools find the best correlation between data set geometric and dose parameters. The goodness of regression may be quantified in terms of R^2 and chi-square. The Model Analytic statistical tool of the RP system was used to evaluate and then exclude potential outliers. These outliers may identify suboptimal plans and/or show a geometry that largely differs from the rest of the training set [19]. All models were tuned following shared procedures among centers. For each modeled OAR, DVHs were analyzed with its regression parameter in

order to exclude possible suboptimal plans that could produce regression parameters 2–3 standard deviations outside the confidence interval. Concerning the goodness of regression models, values of $\chi^2 < 1.1$ and $R^2 > 0.60$ and > 0.4 were considered acceptable in terms of robustness prediction for the ipsilateral lung and the other OARs, respectively [19].

Table S1: Regression parameters of all models.

Institute	*Numb of plans*	*OAR*	*R^2*	*χ^2*	*RP version*
INST. 1	*97*	*IPSI LUNG*	*0.606*	*1.044*	*V 15.6*
		CONTRA LUNG	*0.811*	*1.027*	
		CONTRA BREAST	*0.636*	*1.044*	
		HEART	*0.704*	*1.044*	
INST. 2	*93*	*IPSI LUNG*	*0.741*	*1.014*	*V 16.1*
		CONTRA LUNG	*0.44*	*1.011*	
		CONTRA BREAST	*0.739*	*1.049*	
		HEART	*0.744*	*1.026*	
INST. 3	*79*	*IPSI LUNG*	*0.605*	*1.115*	*V 13.6*
		CONTRA LUNG	*0.558*	*1.045*	
		CONTRA BREAST	*0.446*	*1.042*	
		HEART	*0.441*	*1.078*	
INST. 4	*101*	*IPSI LUNG*	*0.687*	*1.013*	*V 15.6*
		CONTRA LUNG	*0.587*	*1.023*	
		CONTRA BREAST	*0.580*	*1.038*	
		HEART	*0.500*	*1.044*	
INST. 5	*92*	*IPSI LUNG*	*0.686*	*1.065*	*V 13.6*
		CONTRA LUNG	*0.518*	*1.057*	
		CONTRA BREAST	*0.439*	*1.08*	
		HEART	*0.56*	*1.068*	
INST. 6	*100*	*IPSI LUNG*	*0.763*	*1.054*	*V 15.6*
		CONTRA LUNG	*0.662*	*1.031*	
		CONTRA BREAST	*n.a*	*n.a*	

		HEART	*0.758*	*1.019*	
INST. 7	*73*	*IPSI LUNG*	*0.618*	*1.035*	*V 15.5*
		CONTRA LUNG	*0.568*	*1.052*	
		CONTRA BREAST	*n.a*	*n.a*	
		HEART	*n.a*	*n.a*	
INST. 8	*30*	*IPSI LUNG*	*0.655*	*1.142*	*V 16.1*
		CONTRA LUNG	*n.a*	*n.a*	
		CONTRA BREAST	*n.a*	*n.a*	
		HEART	*n.a*	*n.a*	
INST. 9	*68*	*IPSI LUNG*	*0.66*	*1.041*	*V 15.5*
		CONTRA LUNG	*0.541*	*1.068*	
		CONTRA BREAST	*0.643*	*1.033*	
		HEART	*0.766*	*1.021*	
INST. 10	*61*	*IPSI LUNG*	*0.615*	*1.229*	*V 16 .1*
		CONTRA LUNG	*0.709*	*1.118*	
		CONTRA BREAST	*n.a*	*1.086*	
		HEART	*0.638*	*1.086*	

Supplementary Material S3–Planning and clinical goals for VPSRG's test set cohort

The VPSRG patients' cohort was initially planned and treated with TF using manually optimized wedges or FiF techniques, using electronic tissue compensation (eComp) and skin flash. Plans' clinical goals are reported as follows:

**Clinical Goals**	
PTV	D2% < 42.05 Gy
	D90% > 36.05 Gy

	D95% > 38.05 Gy
Heart	V25 Gy < 2%
	Dmean < 3 Gy
Lung_L	V5 Gy < 10%
Lung_R	V16 Gy < 10%
	V5 Gy < 50%
Spinal Canal	Dmax < 40 Gy

Supplementary Material S4—Operative Instructions for external validation data set

- Select 20 patients representative of VPSRG.
- For each patient, set the geometry (following 'geometry setup' instructions).
- For each patient, run models (up to 10 models), track the outside threshold values, and export plan and estimated bands (following 'run model' instructions).

1. Geometry setup

- Open the clinical tangential field TF (3DCRT or FiF) plan of the first patient, create a new course, and copy the clinical plan to the new course.
- Export the dose distribution: 'File DICOM Export Filter'.
- Create a new plan for the PTV with two opposing 60°–220° half-arcs with collimator tilt of 10° (Fig. 1).
- Insert a 'virtual' bolus to the whole body (thickness 1.5cm and HU = -500) and link it to the two arcs (needed to select the correct field sizes accounting for the margin in air).

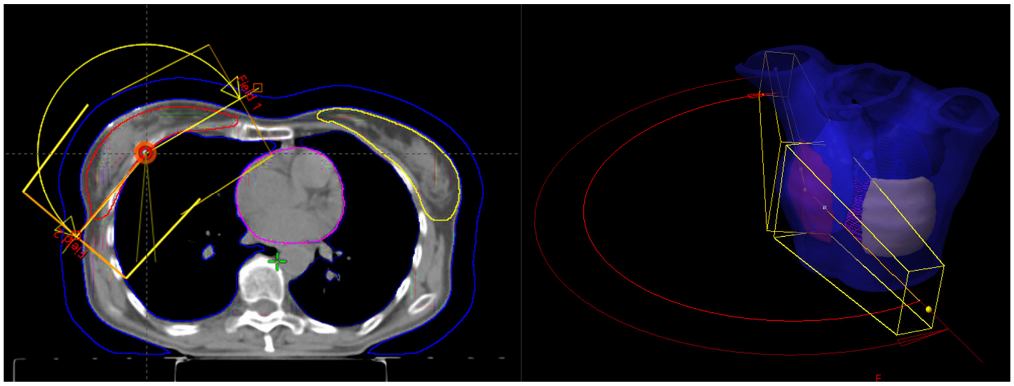

Figure S1 – Beam setup.

- For the first arcs (60-220), display the BEV and set the correct field sizes (Figure S2):
    - o Set a Y margin from the PTV of approx. 1 cm;
    - o For the X margin, display the BEV for the 220° and set the dimensions in X so that the bolus is within the edge of the anterior collimator and the PTV is within the edge of the posterior collimator. In this way also for the other angles in the range 60-40 /240-220 (the possible ‘delivery angles’ 60-40° and 240-220°), the dimensions in X will be respected.
- For the second arc (220-60) the procedure is the same (Figure S2): set the BEV at 40 and select the correct dimensions in X direction.
- It is recommended to save the plan as a template so that it can be applied to other patients, modifying only the dimensions of the fields: 'Planning -> Templates and Clinical Protocols -> Create Plan Template from Plan’.

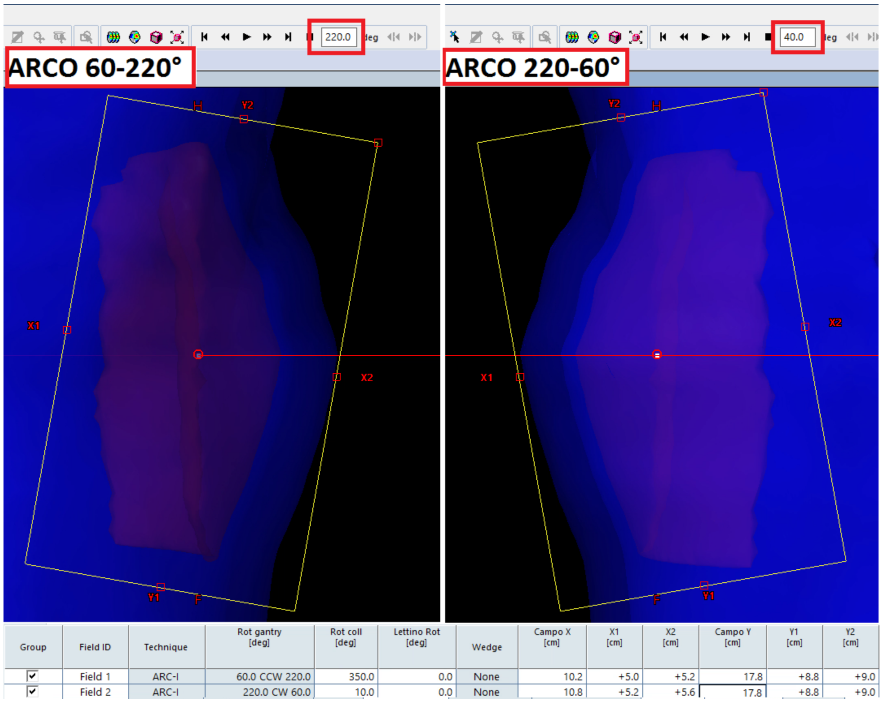

Figure S2 – BEV configuration.

- To link the clinical TF dose to the generated plan, you must first delete the copy of the original plan (if you try to import the dose distribution the system automatically associates it with the plan used for the export). Then: 'File-> Import DICOM Import Filter' -> retrieve the exported dose distribution and link it to the created plan (fig.3): drag the ‘Dose’ icon to the plan and the icon becomes green). Different TPS versions could have a different ‘Import DICOM Imported Filter’ layout.

-

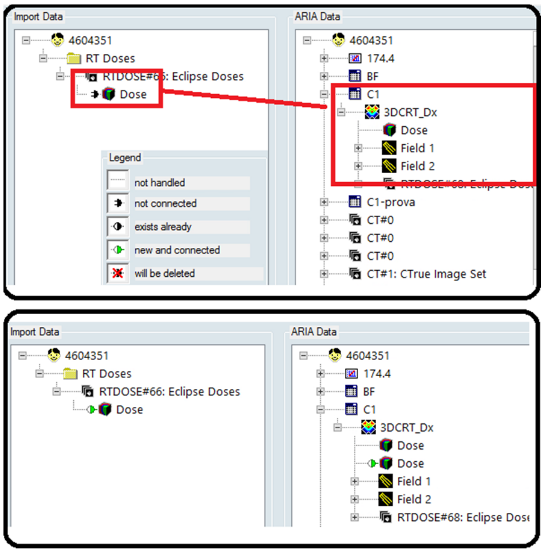

Figure S3 – Link the clinical TF dose to the generated plan.

- You must correct the total prescribed dose and the number of fractions and re-normalize the dose as for the original clinical plan. The system does not set them correctly.

2. Run model

- Make a copy of the arc-plan with the linked TF distribution.

- Open the optimization panel ('Planning-> Optimization -> Optimize …) and load the selected model (‘Estimate DVH -> DVH EStimation Model -> …’), assigning the prescription dose to the PTV according to the original clinical plan.

- Click ‘Generate Estimates and Objectives’.

- If the ‘Estimation Statistics’ appears (fig. 4), mark for each matched structure if it is outside the threshold (using the Excel template ‘template Estimate Statistic’) and for which features.

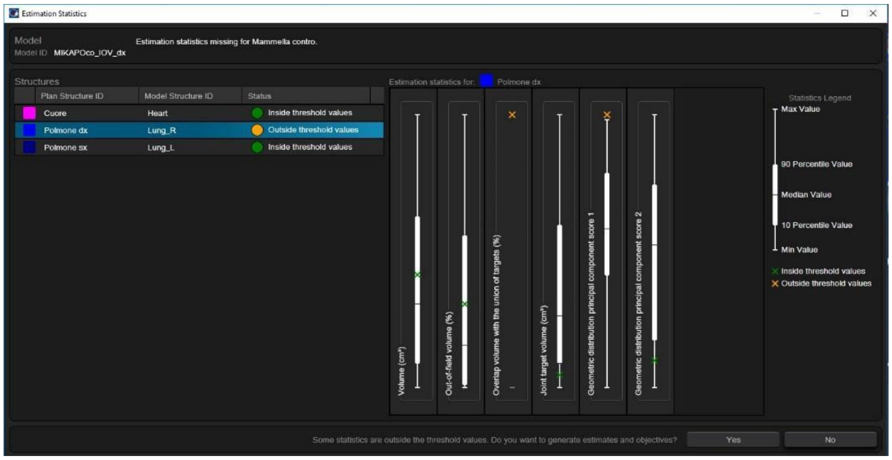

Figure S4 – Estimation statistic.

- Click Yes to obtain the estimated DVHs and then click Ok to return to the external beam panel.

- Export the clinical DVH and the predicted ones (using the script ‘ExportDVH.esapi’ or ‘ExportDVH_v13.6.esapi’ for version 13.6).

- Run the script: ‘Tools → Scripts… → select the file → Run’.

- Select the saving path, match organs, and flag DVH and DVHE. Then, click on ‘Save and Continue’: this allows exporting the clinical DVH and the estimated DVH in terms of minimum and maximum bands (fig. 5).

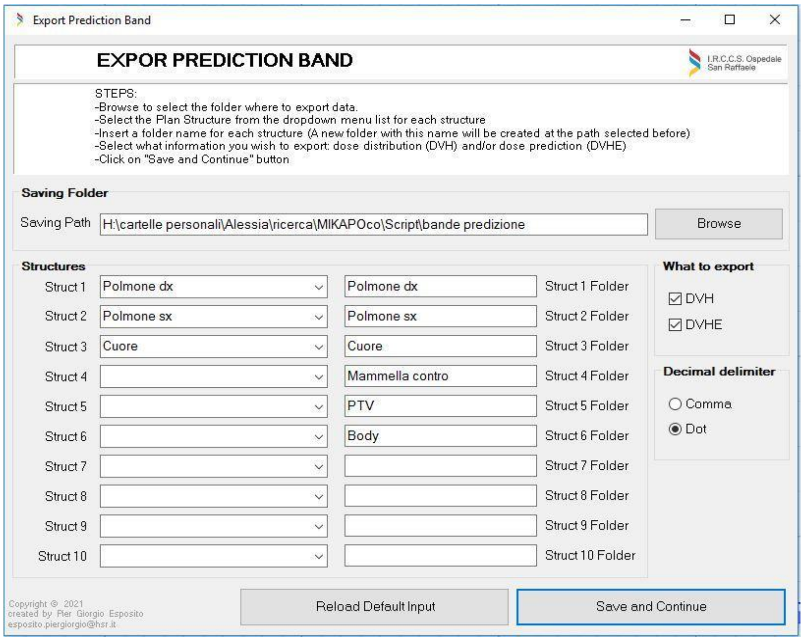

Figure S5 – Export prediction band script interface.

- Folders will be created in the destination path for the various organs in which there are Excel files named as 'id name DVHE organ 0' = minimum volume band in %, 'id name DVHE organ 1' = maximum volume band in %, 'id name DVH organ' = clinical volume DVH in cc. It does not report the percentage of the dose, which is always the same, i.e., from 0 to 100% with steps of 0.1 Gy.

Supplementary Material S5—IPSI lung predicted DVH’s categorization

Regarding inter-consortia transferability, the prediction of ipsilateral lung DVH was further quantified and categorized.
As shown, respectively, in Figures S6, S7, S8, and S9, the following categories have been defined:

- Optimal prediction: cDVH within the predicted DVH band (min–max) for the entire V20%–V80% range (Figure S6);
- Improved prediction: cDVH above the predicted DVH band (min–max) for the entire V20%–V80% range (Figure S7);
- Suboptimal prediction: cDVH below the predicted DVH band (min–max) for the entire V20%–V80% range (Figure S8);
- Failed prediction: predicted DVH band (min–max) strongly disagrees with the cDVH (Figure S9).

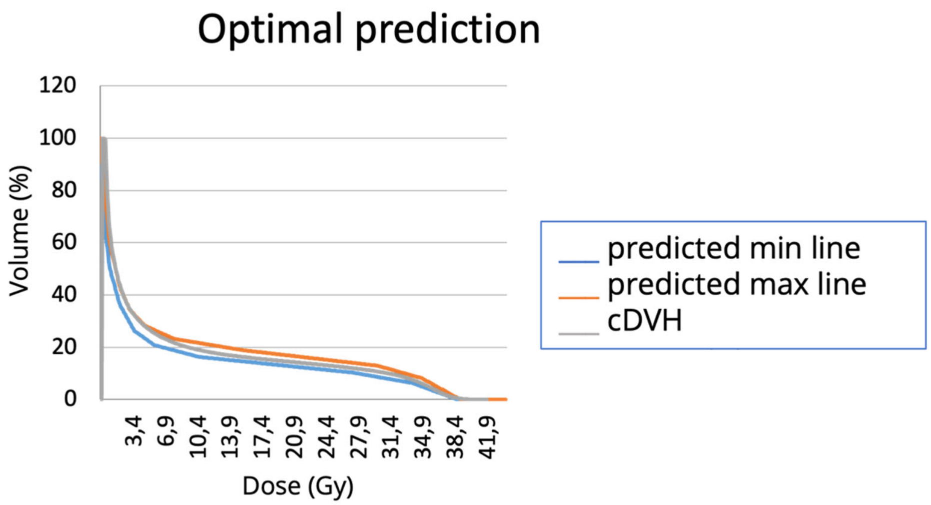

Figure S6—Optimal prediction: cDVH is within the predicted DVH band for the entire V20%-V80% range.

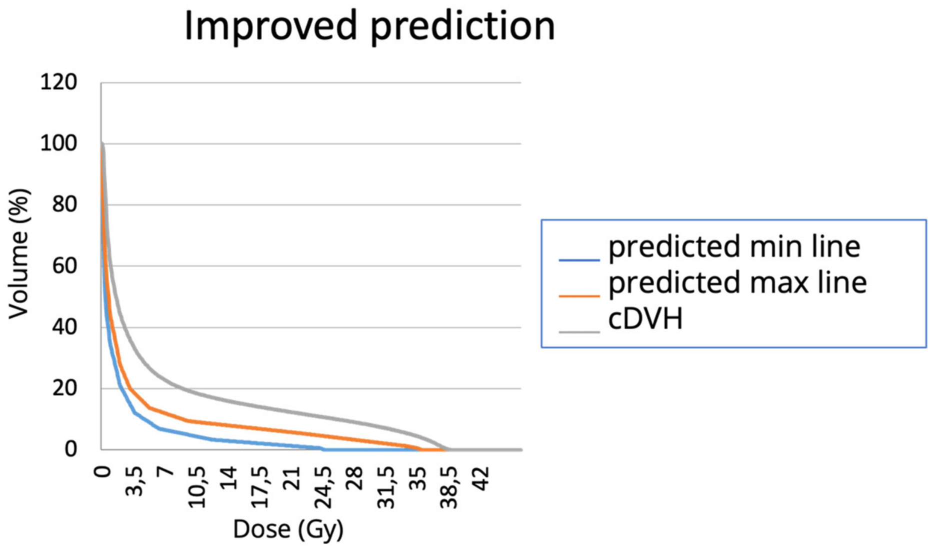

Figure S7—Improved prediction: cDVH above the predicted DVH band for the entire V20%-V80% range.

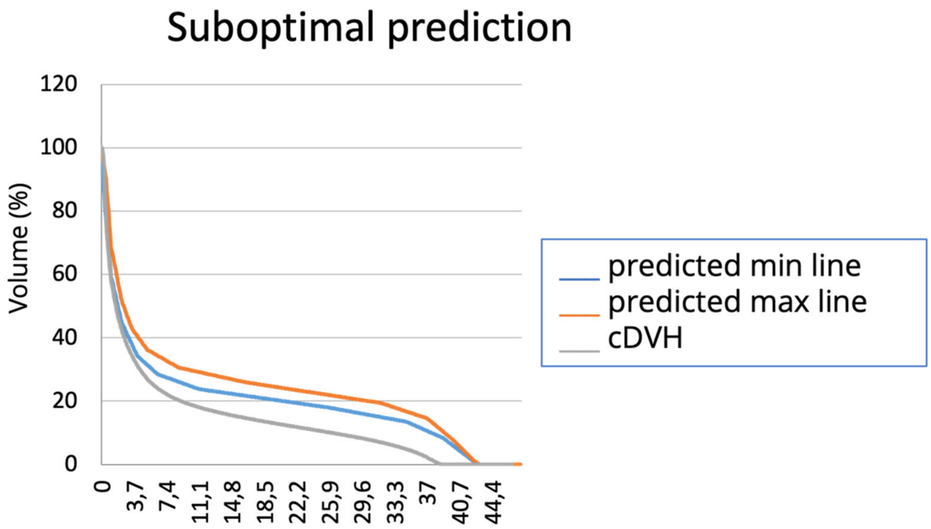

Figure S8—Suboptimal prediction: cDVH below the predicted DVH band for the entire V20%-V80% range.

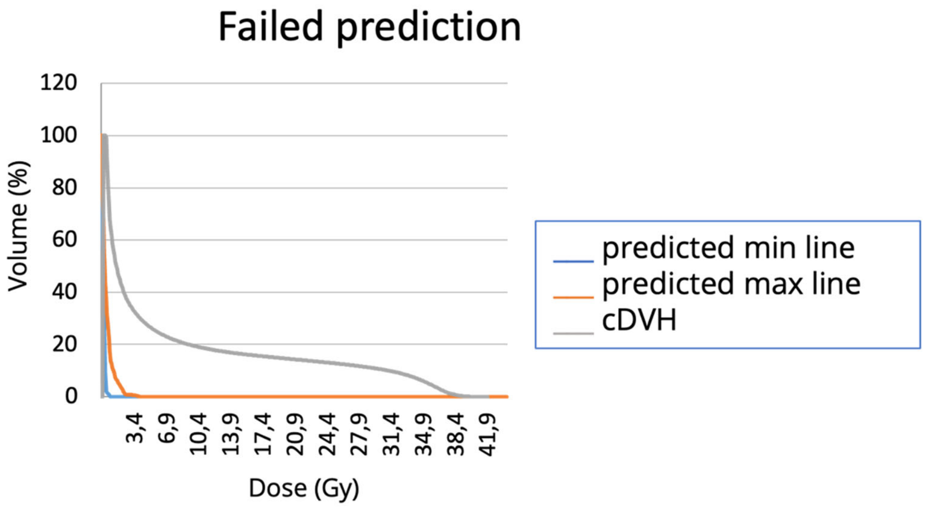

Figure S9– Failed prediction: predicted DVH band strongly disagrees with the cDVH.

Results

In the context of the 20 external cross-validation data set tests conducted by VPSRG, the ipsilateral lung DVH predictions from MIKAPOCo's KB models were categorized and quantified as described before, yielding 76% optimal predictions, 15% improved predictions, 7% suboptimal predictions, and 2% of failed predictions. The results are depicted in the following pie chart (Figure S10).

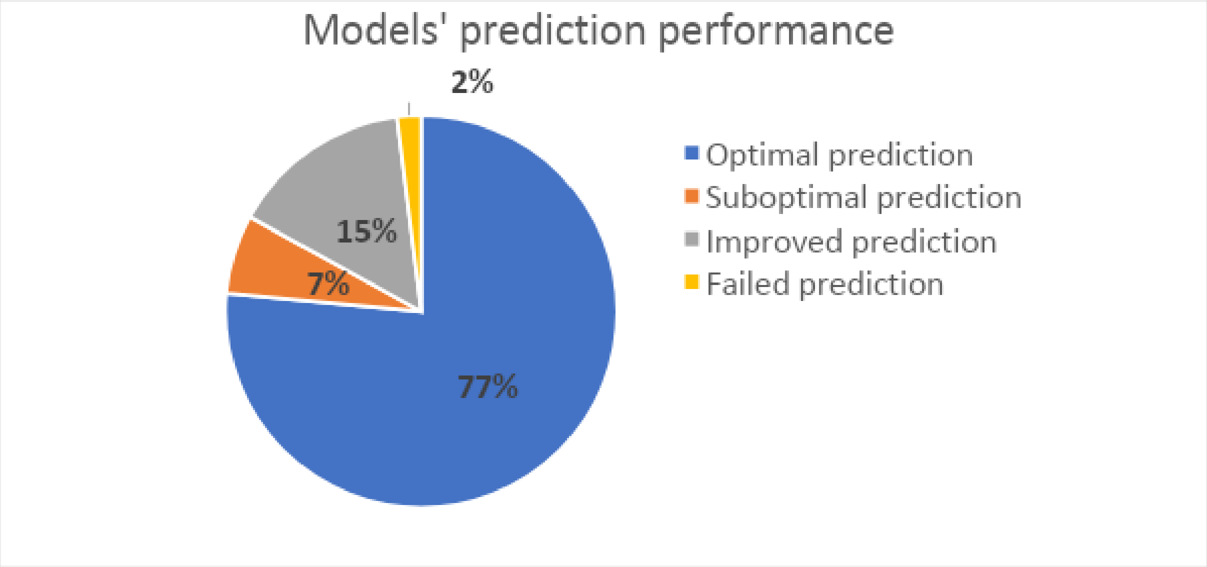

Figure S10—Results of the models' prediction of MIKAPOCo models on VPSRG external cross-validation data test concerning the ipsilateral lung predicted DVHs: categorized into optimal, suboptimal, improved, and failed.

References

45. Berry SL, Ma R, Boczkowski A, Jackson A, Zhang P, Hunt M. Evaluating inter-campus plan consistency using a knowledge based planning model. Radiother Oncol 2016;120:349–55. https://doi.org/10.1016/j.radonc.2016.06.010.

46. Schubert C, Waletzko O, Weiss C, Voelzke D, Toperim S, Roeser A, et al. Intercenter validation of a knowledge based model for automated planning of volumetric modulated arc therapy for prostate cancer. The experience of the German RapidPlan Consortium. PLoS One 2017;12:e0178034. https://doi.org/10.1371/journal.pone.0178034.

47. Ueda Y, Fukunaga J-I, Kamima T, Adachi Y, Nakamatsu K, Monzen H. Evaluation of multiple institutions' models for knowledge-based planning of volumetric modulated arc therapy (VMAT) for prostate cancer. Radiat Oncol 2018;13:46. https://doi.org/10.1186/s13014-018-0994-1.

48. Kamima T, Ueda Y, Fukunaga J-I, Shimizu Y, Tamura M, Ishikawa K, et al. Multi-institutional evaluation of knowledge-based planning performance of volumetric modulated arc therapy (VMAT) for head and neck cancer. Phys Med 2019;64:174–81. https://doi.org/10.1016/j.ejmp.2019.07.004.

49. Kavanaugh JA, Holler S, DeWees TA, Robinson CG, Bradley JD, Iyengar P, et al. Multi-Institutional Validation of a Knowledge-Based Planning Model for Patients Enrolled in RTOG 0617: Implications for Plan Quality Controls in Cooperative Group Trials. Pract Radiat Oncol 2019;9:e218–27. https://doi.org/10.1016/j.prro.2018.11.007.

27. Panettieri V, Ball D, Chapman A, Cristofaro N, Gawthrop J, Griffin P, et al. Development of a multicentre automated model to reduce planning variability in radiotherapy of prostate cancer. Phys Imaging Radiat Oncol 2019;11:34–40. https://doi.org/10.1016/j.phro.2019.07.005.

28. Tudda A, Castriconi R, Benecchi G, Cagni E, Cicchetti A, Dusi F, et al. Knowledge-based multi-institution plan prediction of whole breast irradiation with tangential fields. Radiother Oncol 2022;175:10–6. https://doi.org/10.1016/j.radonc.2022.07.012.

50. Castriconi R, Tudda A, Placidi L, Benecchi G, Cagni E, Dusi F, et al. Inter-institutional variability of knowledge-based plan prediction of left whole breast irradiation. Physica Medica 2024;120:103331. https://doi.org/10.1016/j.ejmp.2024.103331.

19. Castriconi R, Esposito PG, Tudda A, Mangili P, Broggi S, Fodor A, et al. Replacing Manual Planning of Whole Breast Irradiation With Knowledge-Based Automatic Optimization by Virtual Tangential-Fields Arc Therapy. Front Oncol. 2021 Aug 24;11:712423. doi: 10.3389/fonc.2021.712423
51. Esposito PG, Castriconi R, Mangili P, Fodor A, Pasetti M, Di Muzio NG, et al.. Virtual Tangential-fields Arc Therapy (ViTAT) for whole breast irradiation: Technique optimization and validation. Phys Med. 2020 Sep;77:160-168. doi: 10.1016/j.ejmp.2020.08.011. Epub 2020 Aug 28
